# The Effect of Intravenous Dexamethasone and Lidocaine on Propofol-Induced Vascular Pain: A Randomized Double-Blinded Placebo-Controlled Trial

**DOI:** 10.1155/2013/734531

**Published:** 2013-07-15

**Authors:** Shireen Ahmad, Gildasio S. De Oliveira, Paul C. Fitzgerald, Robert J. McCarthy

**Affiliations:** Department of Anesthesiology, Northwestern University Feinberg School of Medicine, 251 East Huron Street, Suite F5-704, Chicago, IL 60611, USA

## Abstract

*Background.* The mechanism for pain associated with intravenous administration of propofol is believed to be related to the release of nitric oxide. We hypothesized that pain following propofol injection would be reduced by pretreatment with dexamethasone. *Methods.* One hundred fourteen female subjects received 5 mL of preservative-free saline, 0.5 mg**·**kg^−1^ of lignocaine hydrochloride 10 mg**·**mL^−1^ or 0.25 mg**·**kg^−1^ of dexamethasone, intravenously, following exsanguination and occlusion of the veins of the arm. This was followed by a 0.5 mg**·**kg^−1^ injection of propofol. Pain scores, facial grimacing, arm withdrawal, and vocalization were recorded prior to and at 15 and 30 seconds following the injection of propofol. *Results.* The incidence of moderate to severe pain following the injection of propofol was significantly decreased with both lidocaine and dexamethasone. Hand withdrawal was also significantly decreased in comparison to saline. *Conclusion.* Low dose dexamethasone is commonly used as an antiemetic, and, in larger doses, it has been demonstrated to provide prolonged postoperative analgesia. At higher analgesic doses, dexamethasone may also reduce pain associated with the injection of propofol. This effect is probably related to the effect of the steroid on nitric oxide production associated with intravenous propofol injection.

## 1. Introduction

Propofol (Diprivan, 2,6-di-isopropylphenol) was introduced into clinical use in 1986 and has now become the most widely used intravenous anaesthetic, despite the high incidence of localized pain on injection. In studies of propofol injection into an intravenous catheter in a forearm cephalic vein or vein on the back of the hand, the incidence of pain was 68%–72% [1, 2]. The pain is immediate and can be profound, and it has been ranked the seventh out of the 33 anesthesia outcomes deserving high priority for improvement, by a panel of anaesthesiologists from academic and community practices [3]. In addition, the hyperdynamic cardiovascular response to the pain can precipitate adverse events in high-risk patients with history of coronary artery disease and/or abnormal heart rhythm.

Clinical strategies designed to alleviate propofol-related pain have been described in the literature including cooling the extremity, dilution of the propofol solution injecting propofol into a large antecubital vein, and the application of topical nitroglycerin on the skin overlying the tip of the intravenous catheter. Injection of lidocaine to prevent propofol injection pain is the most extensively studied technique and is commonly used in clinical practice but with variable results [4]. Our group has recently demonstrated that dexamethasone is an effective strategy to minimize postoperative pain and PONV [5, 6]. In addition, the administration of intravenous dexamethasone has not been associated with increased incidence of infection or altered hyperglycemic response in the perioperative period [7, 8]. However, it remains to be determined whether the preoperative administration of intravenous dexamethasone can also reduce the incidence of pain on injection of propofol, which would justify the administration of the drug before anesthetic induction.

Propofol has been shown to release nitric oxide (NO) from vessels in animals and humans, and the release of nitric oxide has been linked to the generation of pain in the veins in humans [9–11]. The effects of corticosteroids such as dexamethasone on NO production have been previously demonstrated [12, 13]. In addition, the efficacy of steroids to alter nitric oxide release has also been demonstrated in several disease conditions [14, 15]. Therefore, the choice of dexamethasone to minimize propofol-induced vascular pain was not only based on its wide clinical utilization but also due to its biological basis.

The purpose of this study was to compare pain scores and behavioral signs of discomfort among groups pretreated with high-dose dexamethasone, lidocaine, or saline that received a propofol bolus injection. We hypothesize that subjects receiving dexamethasone would have less discomfort than the ones receiving saline.

## 2. Materials and Methods

Following the approval by the Institutional Review Board of Northwestern University, informed consent was obtained from adult subjects who were older than 18 years, ASA PS I & II, and scheduled to undergo outpatient gynaecologic surgery under general anaesthesia. Subjects were excluded if they had a hypersensitivity to propofol or soy bean oil, glycerol, egg lecithin, or sodium oleate, if they had small caliber veins on the dorsum of the hands, if they required intravenous drug administration prior to induction of anaesthesia, or if they required a rapid sequence induction of anaesthesia. Pregnant or lactating patients and those with a history of chronic pain, with neurologic, psychiatric, significant cardiac, renal, or liver disease, or taking sedatives or analgesics preoperatively were excluded.

Subjects were randomly assigned (computer-generated table) to one of the following three pretreatment groups: preservative-free, saline, 0.5 mg*·*kg^−1^ of lignocaine hydrochloride 10 mg*·*mL^−1^ or 0.25 mg*·*kg^−1^ of dexamethasone sodium phosphate. The study drug was diluted with preservative-free saline to a final volume of 5 mL. Prior to the transference to the operating room, an 18-gauge intravenous cannula was inserted into the largest vein on the dorsum of the nondominant hand, and an infusion of Lactated Ringer's solution was started at a rate of 5 mL*·*kg*·*hr^−1^. In addition, an appropriately sized blood pressure cuff was placed on the upper arm above the intravenous site. Subjects were asked to rate the severity of pain experienced on insertion of the IV cannula using a visual analogue scale (VAS) and a verbal rating score (VRS) for pain (0 = no pain, 1 = mild pain, 2 = moderate pain, and 3 = severe pain). No other drugs were administered through the IV cannula prior to the administration of the study drugs. The infusion was stopped, and the arm was elevated for 15 seconds. The blood pressure cuff was inflated by activating the “Start venous stasis” button on the physiological monitor (Datex-Engstrom, Helsinki, Finland), and the pretreatment study drug was injected (5 s) into the injection port closest to the cannulation site. The study medication was prepared by a single investigator (Paul C. Fitzgerald), and the investigator who administered the study drugs was blind to the study group. The patient was then asked to rate the discomfort associated with the injection using the VRS scale. Behavioral signs (facial grimacing, arm withdrawal, and vocal response) were also recorded following the injection.

Two minutes following the study drug administration, the blood pressure was deflated by activating the “Stop venous stasis” button, and the intravenous infusion was restarted by releasing the roller clamp. The 0.5 mg*·*kg^−1^ propofol was injected through the same injection port over 5 seconds. Spontaneous complaints of pain were noted. If there were none, 15 and 30 seconds following the injection the patient was asked to rate the discomfort (VRS) associated with the propofol injection. Behavioral signs were also recorded. After the last recording, anaesthesia was induced in the standard manner, and anesthetic management was at the discretion of the anaesthesiologist. Subjects were contacted 24 h following surgery and questioned about pain or swelling at the injection site.

## 3. Statistical Analysis

The primary outcome variable was the incidence of moderate to severe pain reported. Based on the review of the literature, we anticipated that 65 percent of those treated with saline, 30 percent of those treated with lignocaine, and 40 percent of those treated with dexamethasone would report moderate to severe pain. Assuming these rates of occurrence, an effect size (*W*) of 0.29 was calculated. A sample size of 115 achieves 80% power to detect a significant difference among groups at “alpha” of 0.05 using the two-degree freedom *χ* ^2^ test. Clinical characteristics among the groups were compared using one-way ANOVA or the Kruskal-Wallis *H* test. The incidence of moderate/severe pain and the number of patients reporting pain following propofol injection, the incidence of behavioral signs, and other adverse effects were compared using a chi-square analysis. Estimates of exact *P* values were determined for the Mann-Whitney and the *χ* ^2^ test using the Monte Carlo method with 10,000 samples and confidence limits of 99%. All tests are reported as two sided, and a *P* < 0.05 was required to reject the null hypothesis. Nominal and categorical data are presented as counts and percentage of respondents. Interval data are presented as medians with interquartile range (IRQ). All reported *P* values are two tailed. Data were analyzed using NCSS 2007 version 7.1.20, release date 2/19/2010, NCSS LLC, Kaysville, UT, USA, and PASW Statistics 18.0.2, release date 4/2/2010, SPSS, Inc., Chicago, IL, USA.

## 4. Results

One hundred and twenty-two subjects were enrolled. Eight subjects were excluded from the study prior to randomization: 1 due to a changed anaesthetic plan, 4 due to cancellation of the procedure, 2 due to pain prior to the study drug administration at the intravenous site, and 1 because a nonstudy drug was administered. The median age of the subjects was 37 years (interquartile range: 32 to 42 y), and weight was 65 kg (interquartile range: 55 to 77 kg) and did not differ among groups.

All subjects were awake and alert during the period of pain assessment following injection of propofol. The median VAS for reported pain during IV cannula insertion was 2 (interquartile range: 0 to 6.6) in the saline group, 5 (1 to 10) in the lignocaine group, and 3 (0 to 13.5) in the dexamethasone group (*P* = 0.20). The mean (SD) doses (mg) of propofol administered to the saline, lignocaine, and dexamethasone groups were the following: 30.8 ± 3.6, 30.2 ± 2.9, and 31.2 ± 3.3 (*P* = 0.48), respectively.

The incidence of moderate to severe pain at 15 s after the injection of propofol was 56% in the saline group, 14% in the lignocaine group, and 24% in the dexamethasone group (*P* = 0.002), and the incidence of moderate to severe pain at 30 s after the injection of propofol was 60% in the saline group, 26% in the lignocaine group, and 41% in the dexamethasone group (*P* = 0.009) (Table 1). At 15 s, dexamethasone reduced the number of subjects reporting moderate to severe pain (*P* = 0.03) as well as exhibiting hand withdrawal (*P* = 0.005) compared to saline. Dexamethasone also reduced the number of subjects who exhibited grimacing (*P* = 0.02) and hand withdrawal (*P* = 0.02) compared to saline at 30 s. Lignocaine and dexamethasone did not differ in the reported pain or behavioral signs at either time.

Twenty-four hours postoperatively, 2 subjects reported mild pain in the study arm (1 saline and 1 lignocaine), and 1 subject (lignocaine) reported swelling at the catheter site. No other complications were noted.

## 5. Discussion

The most important finding of this study was the reduction in the number of subjects that reported moderate or severe pain following propofol administration when pretreated with dexamethasone compared to saline. Systemic dexamethasone has been commonly used perioperatively to minimize postoperative nausea and vomiting and to improve overall quality of recovery [5, 6]. In addition, dexamethasone has been shown to decrease nitric oxide production which has been shown to mediate propofol-induced vascular pain [11–13]. The current study suggests that the preoperative administration of dexamethasone also diminishes pain on propofol injection.

The need to treat propofol-induced nociception is essential not only because it is unpleasant, but also because it can lead to serious sequelae such as myocardial ischemia when hemodynamic changes occur in response to the pain associated with injection [16]. The reduction in propofol-induced pain behaviors was also achieved with lidocaine in the current investigation. Nevertheless, dexamethasone has an advantage over lidocaine to improve postoperative quality of recovery since it does not require the additional administration of an intraoperative infusion [17].

Studies suggest that the mechanisms underlying nociception from vascular tissues following propofol injection are multifactorial in origin. Propofol has been demonstrated *in vitro* to stimulate nitric oxide (NO) release [18]. Nociceptive nerve endings have been found in the endothelium of veins in humans, a well-known source of NO, suggesting a role of NO in nociception [19, 20]. In addition, NO from the vascular endothelium binds to guanyl cyclase which catalyzes the conversion of guanosine triphosphate to guanosine monophosphate, which facilitates PGE2-induced hyperalgesia [11]. It has been found that pain following intravenous injection of bradykinin and hyperosmolar solutions can be blocked by pretreatment with NO synthase (NOS) inhibitor, suggesting that an intact NOS pathway is needed to elicit vascular nociception [21].

Previous investigators have described various attempts to eliminate propofol-induced vascular pain [22]. Many of these studies lack a scientific rationale, and the pharmacologic interventions often tested do not have a biologic basis. The effects of anesthetics and perioperative stress on NO production have been previously demonstrated [23, 24]. In patients with asthma, corticosteroids reduced the levels of exhaled NO, and dexamethasone inhibited nitrite production in cells from the human joint and lung epithelial cells [12]. Dexamethasone is commonly used perioperatively as an antiemetic in both adults and children in doses ranging from 150 *µ*g/kg to 0.5 mg/kg [25, 26].

There are limitations to our study design that may have affected our findings. We used a single dose of dexamethasone (0.25 mg/kg) which is greater than the dose commonly used as an antiemetic, but it is comparable to doses that have been shown to provide a prolonged postoperative analgesic effect without adverse effects. Furthermore, it has been noted that patients receiving dexamethasone 20 mg daily for five days to control chemotherapy-induced nausea and vomiting had no evidence of immunosuppression or hypothalamic-pituitary-adrenal axis dysfunction [27]. A limitation of all studies evaluating vascular pain following propofol administration is the use of a subhypnotic dose of propofol so that reliable pain assessments reporting can be obtained. Finally, our study was underpowered to detect the difference in the incidence of moderate to severe pain between lidocaine and dexamethasone.

In conclusion, the findings of this study suggest the effectiveness of dexamethasone pretreatment in reducing vascular pain following propofol administration. Pretreatment with dexamethasone was more effective than saline and had a similar efficacy as lidocaine prior to propofol injection. Since dexamethasone is commonly used to prevent postoperative nausea and vomiting and to improve post-surgical recovery, clinical practitioners should consider using dexamethasone preoperatively to minimize propofol-induced vascular pain and its undesirable side effects.

## Figures and Tables

**Table 1:** Pain and behavioral responses following propofol administration.

	Saline (*n* = 37)	Lidocaine (*n* = 43)	Dexamethasone (*n* = 34)	*P *	Saline (*n* = 37)	Lidocaine (*n* = 43)	Dexamethasone (*n* = 34)	*P *
Time (s)	15				30			
Moderate/severe pain *n* (%)	18 (56)	6 (14)	8 (24)	0.002	22 (60)	11 (26)	14 (41)	0.009
Pain intensity (*n*)								
None	13	30	22	0.03	5	24	15	0.01
Mild	6	7	4		10	8	5	
Moderate	7	2	3		15	8	10	
Severe	11	4	5		7	3	4	
Facial grimacing *n* (%)	0	1 (2)	2 (6)	0.29	5 (14)	3 (7)	0 (0)	0.07
Hand withdrawal *n* (%)	17 (46)	3 (7)	5 (15)	<0.005	21 (58)	5 (12)	10 (29)	<0.005
Crying *n* (%)	1 (3)	0 (0)	1 (3)	0.53	0 (0)	1 (2)	0 (0)	0.44
